# Potential of isotoxic dose escalation with robustly optimized VMAT and IMPT for large hepatocellular carcinoma according to baseline liver function

**DOI:** 10.1002/acm2.70846

**Published:** 2026-10-06

**Authors:** Elise Cherek, Mark Ka Heng Chan, Diane Schott

**Affiliations:** ^1^ Physics Department University of Nebraska Lincoln Lincoln Nebraska USA; ^2^ Department of Radiation Oncology University Nebraska Medical Center Omaha Nebraska USA

**Keywords:** dose escalation, hepatocellular Carcinoma; Liver baseline function, intensity‐modulated proton and photon therapy, robust optimization and evaluation

## Abstract

**Background:**

Large hepatocellular carcinoma (HCC) adjacent to gastrointestinal (GI) organs presents a constrained radiotherapy problem because dose escalation is limited by both hepatic reserve and serial‐organ tolerance. Although intensity‐modulated proton therapy (IMPT) can reduce uninvolved liver irradiation, its achievable advantage over volumetric modulated arc therapy (VMAT) may depend on baseline liver function and robustness to treatment uncertainties.

**Purpose:**

To present and demonstrate a robust isotoxic dose‐escalation framework for evaluating IMPT and VMAT in large HCC adjacent to GI organs while accounting for baseline liver function and treatment uncertainty.

**Methods:**

Thirteen patients with HCC (equivalent tumor diameter, 3.0–20.9 cm) underwent comparative IMPT and VMAT treatment planning. Robust optimization incorporated 5‐mm setup uncertainty for both modalities and 3% range uncertainty for IMPT. Dose escalation was performed using a fixed dose per fraction of 3.87 Gy while varying the maximum acceptable number of fractions, *N*_max_, under non‐cirrhotic, Child‐Pugh A (CP‐A), and Child‐Pugh B (CP‐B) liver‐function assumptions. Liver mean dose constraints were evaluated on nominal dose, while serial‐organ constraints were assessed using both nominal dose and voxel‐wise maximum dose across uncertainty scenarios. Unirradiated liver volume normalized to standard liver volume (ULV/SLV) was used to estimate the relative hazard of radiation‐induced liver disease (RILD).

**Results:**

In the nominal‐dose analysis, IMPT achieved a higher *N*_max_ than VMAT in 5 of 13, 8 of 13, and 8 of 13 patients under non‐cirrhotic, CP‐A, and CP‐B assumptions, respectively, with mean Δ*N*_max_ values of 0.38, 1.23, and 1.69 fractions. Similar trends were observed in the voxel‐wise maximum‐dose analysis, where mean Δ*N*_max_ values were 0.00, 1.08, and 1.77 fractions, respectively. VMAT dose escalation was predominantly limited by mean liver dose, whereas IMPT was more frequently constrained by GI maximum‐dose limits, indicating a shift from liver reserve to serial‐organ tolerance as the primary limiting factor. Among patients with an IMPT dose‐escalation advantage, IMPT maintained higher ULV/SLV and correspondingly lower estimated RILD hazard despite treatment escalation.

**Conclusions:**

IMPT provides greater isotoxic dose‐escalation potential than VMAT for selected patients with large HCC, particularly when impaired baseline liver function causes photon therapy to be limited by mean liver dose. The proton advantage is ultimately constrained by robust GI organ‐at‐risk tolerances, emphasizing the importance of integrating baseline liver function, liver reserve, and uncertainty‐aware serial‐organ evaluation into modality selection and treatment planning.

## 1 INTRODUCTION

Large hepatocellular carcinoma (HCC) adjacent to gastrointestinal (GI) organs presents a constrained radiotherapy problem: tumor dose escalation must be balanced against residual hepatic reserve and serial‐organ tolerance. This balance is particularly difficult in patients with underlying liver disease, where baseline liver function may determine the safe prescription as much as tumor size or geometry.^1^, ^2^, ^3^

The ASTRO clinical practice guideline supports external beam radiotherapy for appropriately selected patients with primary liver cancer and emphasizes a risk‐adaptive, individualized approach to dose and fractionation based on tumor extent, organ‐at‐risk proximity, and baseline liver function.^4^ In this context, the clinically relevant question is not only whether a plan can meet a fixed prescription, but how far dose can be escalated while respecting liver‐function‐specific and serial‐organ constraints.

Intensity‐modulated proton therapy (IMPT) may improve this therapeutic window by reducing low‐ and intermediate‐dose exposure to uninvolved liver compared with photon therapy. Proton‐specific RILD studies further suggest that unirradiated liver volume normalized to standard liver volume (ULV/SLV) provides liver‐reserve information beyond mean liver dose (MLD) alone.^5^, ^6^ However, the benefit of liver sparing may be limited when large tumors abut stomach, duodenum, bowel, or heart, where focal maximum‐dose constraints can become the dominant barrier to escalation.^7^

Prior proton‐versus‐photon comparisons in HCC have used dosimetric, model‐based, iso‐TCP, or iso‐NTCP approaches, but many were performed at fixed prescription levels, relied on nominal or PTV‐based evaluation, or did not fully address uncertainty‐sensitive GI constraints.^8^, ^9^, ^10^, ^11^, ^12^, ^13^, ^14^, ^15^, ^16^ For large HCC near GI mucosa, such comparisons may bias modality selection because setup and range uncertainties can materially affect near‐maximum serial‐organ dose.

We therefore evaluated IMPT and VMAT using an isotoxic robust dose‐escalation framework for large HCCs near GI organs. Dose escalation was assessed separately under non‐cirrhotic, Child‐Pugh A, and Child‐Pugh B liver‐function assumptions using a fixed dose per fraction and variable maximum fraction number, *N*_max_. Serial‐organ constraints were evaluated using both nominal dose and voxel‐wise maximum dose across uncertainty scenarios, and ULV/SLV was used to estimate residual RILD hazard at the respective modality‐specific *N*_max_.

## 2 MATERIALS AND METHODS

### 2.1 Patient selection and imaging

Thirteen HCC cases were included in this planning analysis. The equivalent tumor diameter ranged from 3.0 to 20.9 cm, and tumor volume ranged from 14.6 to 4768.5 cm^3^ (median 526.2 cm^3^; equivalent spherical diameter: 10.2 cm). The dataset included the large‐tumor cohort from prior hypofractionated liver radiotherapy planning work together with additional cases used to extend the tumor‐size range for the present isotoxic analysis.

Gross tumor volume (GTV) was contoured on triphasic contrast‐enhanced CT fused with breath‐hold CT acquired under active breathing control. The uninvolved liver volume (LV) was defined as whole liver minus GTV. Unirradiated LV (ULV) was defined as the absolute LV receiving < 1 Gy. The clinical target volume (CTV), normal liver, stomach, duodenum, bowel, spinal cord, heart, and other relevant OARs were used for treatment planning and plan evaluation.

### 2.2 Robust proton and photon treatment planning

All plans were generated on the breath‐hold CT in RayStation version 11B (RaySearch Laboratories, Sweden). A common robust‐planning framework was used for both IMPT and VMAT. The clinical target‐coverage goal was voxel‐wise minimum CTV V94% ≥ 98%, where the voxel‐wise minimum dose was defined as the lowest dose to each voxel across the nominal and uncertainty scenarios.

Spot‐scanning IMPT plans were generated for an IBA ProteusPlus system with maximum energy of 230 MeV and an in‐air spot size of 7–8 mm at isocenter. Plans used two to three fields selected to minimize tissue heterogeneity along the beam path, avoid unstable body surfaces, and limit overlap of entrance dose. A 4‐cm water‐equivalent range shifter was used for shallow targets with a minimum air gap of 5 cm. Beam‐specific minimum‐dose constraints of 35% and 23% of the prescription dose were used for two‐ and three‐field arrangements, respectively, to limit modulation. A Monte Carlo dose engine was used for optimization and final dose calculation with 0.5% relative statistical uncertainty. All doses were calculated with a constant relative biological effectiveness (RBE) of 1.1.

VMAT plans were generated using 6‐ and 10‐MV flattening‐filter‐free beams from a Varian TrueBeam. Each plan used two to three non‐coplanar partial arcs to maximize CTV coverage while sparing normal liver and other OARs.

Dose inhomogeneity within the CTV was intentionally permitted for both VMAT and IMPT, provided that robust target coverage and all applicable OAR constraints were satisfied. A maximum dose of up to 160% of the prescription dose was allowed within the CTV for VMAT, while no explicit CTV maximum‐dose objective was imposed for IMPT. Thus, target dose homogeneity was not used as a dose‐escalation endpoint, allowing optimization priority to be directed toward robust target coverage and maximal normal‐tissue sparing. This approach is consistent with the heterogeneous dose distributions commonly used in stereotactic body and hypofractionated radiotherapy.

Robustness was modeled with 5‐mm setup uncertainty for both modalities and 3% range uncertainty for IMPT, consistent with reported intra‐ and interfraction liver motion under breath hold and contemporary proton robustness practice.^17^, ^18^, ^19^ In addition to the voxel‐wise minimum CTV coverage goal, robustness was imposed on near‐maximum doses to serial GI tract organs, spinal cord, and heart.

### 2.3 Plan evaluation of isotoxic dose‐escalation strategy

After clinically acceptable robust IMPT and VMAT plans were generated, isotoxic dose escalation was evaluated separately for non‐cirrhotic, CP‐A, and CP‐B liver baselines (Figure 1). A fixed dose of 3.87 Gy per fraction was used over a candidate range of 6–15 fractions, with the search initialized at *N* = 8. At each candidate fraction number *N*, liver MLD was evaluated on the nominal dose against the constraint corresponding to the assumed liver baseline. Serial and near‐serial OAR limits for the GI tract, spinal cord, and heart were evaluated using nominal dose in the nominal‐dose analysis and voxel‐wise maximum dose in the robustness analysis. The voxel‐wise maximum dose was defined as the highest dose to each voxel across the nominal and uncertainty scenarios.

**FIGURE 1:**
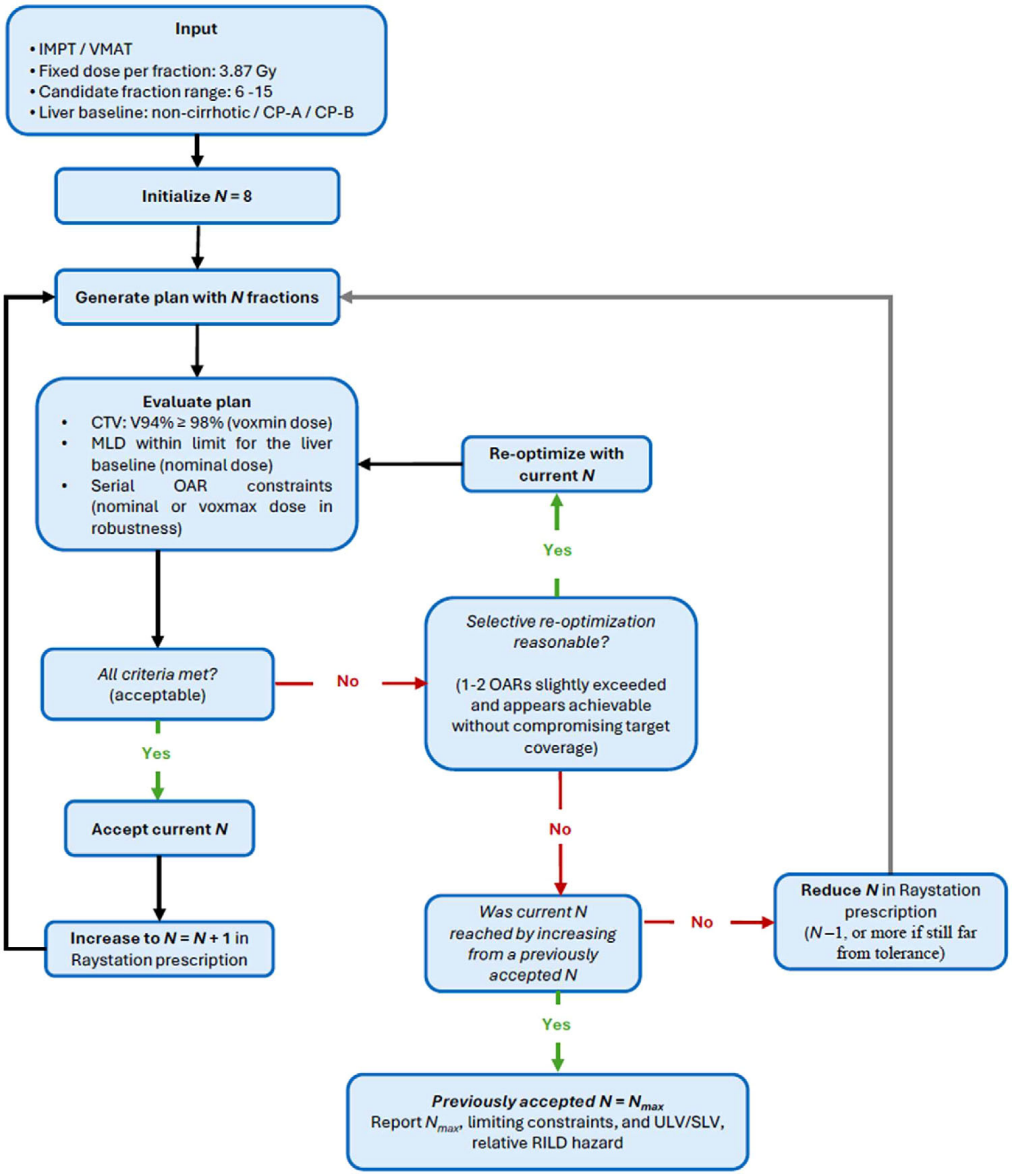
Schematic flowchart of the isotoxic dose‐escalation workflow. IMPT, intensity‐modulated proton therapy; VMAT, volumetric‐modulated arc therapy; CTV, clinical target volume; MLD, mean liver dose; OAR, organ at risk; ULV, uninvolved liver volume receiving < 1 Gy; SLV, standard liver volume; RILD, radiation‐induced liver disease.

If all acceptance criteria were met, *N* was increased by one by adjusting the number of fractions directly in the RayStation prescription to rescale the optimized dose distribution to the corresponding total prescription. The evaluation was repeated until one or more constraints were exceeded. If *N* = 8 failed, lower fraction numbers were evaluated until an acceptable schedule was identified.

When a candidate schedule failing one or two OAR constraints after increasing or decreasing *N*, re‐optimization was performed only when those constraints appeared achievable by trading some dose to other OARs that remained well within tolerance, without compromising robust target coverage. This process was applied to both IMPT and VMAT near the escalation boundary. *N*_max_ was defined as the highest integer fraction number for which an acceptable plan could be achieved.

Normal‐tissue constraints specified for a 15‐fraction schedule were converted to each candidate fraction number using BED equivalence, assuming an alpha/beta ratio of 3 Gy for normal tissue. For the 15‐fraction moderate‐hypofractionation reference schedule, the ASTRO guideline MLD constraints for uninvolved liver were < 24 Gy for non‐cirrhotic liver, < 20 Gy for CP‐A liver, and < 16 Gy for CP‐B7 liver.^4^ Serial‐organ constraints were based on the hypofractionated liver proton regimen reported by Hong et al.^7^ and aligned with the corresponding moderate‐hypofractionation ASTRO constraints, including stomach D0.03 cc < 42 Gy and duodenum, small bowel, and large bowel D0.03 cc < 45 Gy at 15 fractions.

Because the study endpoint was isotoxic escalation rather than comparison at a common prescription, the final accepted *N*_max_ was patient‐, liver‐baseline‐, and modality‐specific. Consequently, absolute dose metrics at the respective *N*_max_ values were not pooled across patients, as these would represent different total prescription doses and different dose‐limiting conditions. Comparative reporting therefore focused on *N*_max_, *∆N*_max_, the dose‐limiting constraint, and liver‐reserve metrics at the modality‐specific *N*_max_.

For each patient and liver baseline, *∆N*_max_ was calculated as the difference in maximum acceptable fraction number between IMPT and VMAT for both nominal‐dose and voxel‐wise maximum‐dose evaluations. At the respective modality‐specific *N*_max_, nominal ULV/SLV was used to estimate the relative RILD hazard of IMPT versus VMAT. Given the published hazard ratio for RILD was 0.902 per 1 percentage‐point increase in ULV/SLV,^5^ the relative RILD hazard for IMPT compared with VMAT was calculated as:

(1) $$\begin{array}{cc} & \mathrm{HR}\left(\mathrm{IMPT}/\mathrm{VMAT}\right)=\exp[\ln\left(0.902\right)\times 100\\ & \;\times ({\left(\mathrm{ULV}/\mathrm{SLV}\right)}_{\mathrm{IMPT}}-{\left(\mathrm{ULV}/\mathrm{SLV}\right)}_{\mathrm{VMAT}}))] \end{array}$$

The percentage reduction in estimated RILD hazard for IMPT relative to VMAT was then calculated as:

(2) $$\begin{array}{cc} & \text{Hazard reduction}(\mathrm{IMPT}/\mathrm{VMAT})\\ & =[1-\mathrm{HR}(\mathrm{IMPT}/\mathrm{VMAT})]\times 100\% \end{array}$$

A positive value indicates lower estimated RILD hazard with IMPT, whereas a negative value indicates higher estimated hazard with IMPT. This calculation provides a relative hazard estimate based on ULV/SLV difference and was not interpreted as an absolute individualized probability of RILD. Individual patient‐level values are provided in Table S1.

Because individual patient sex, height, and weight were not available, ULV/SLV was calculated using reference male and female SLV assumptions of 1599.7 and 1294.4 cm^3^, respectively. Ratios were not truncated in the primary analysis because reference SLV is a population‐level normalization and may be smaller than an individual patient's measured spared liver volume. Liver‐reserve thresholds were summarized using ULV/SLV > 50% for CP‐A and > 60% for CP‐B.

## 3 RESULTS

### 3.1 Achievable maximum fraction number

The equivalent tumor diameter ranged from 3.0 to 20.9 cm, and tumor volume ranged from 14.6 to 4768.5 cm^3^. For patient‐level visualization, patients were ranked from 1 to 13 by increasing equivalent tumor diameter.

In the nominal‐dose analysis, IMPT achieved a higher maximum fraction number, *N*_max_, than VMAT in 5 of 13, 8 of 13, and 8 of 13 patients under the non‐cirrhotic, CP‐A, and CP‐B assumptions, respectively (Figure 2). The corresponding mean ∆*N*_max_, defined as *N*_max_,_IMPT_—*N*_max_,_VMAT_, was 0.38, 1.2, and 1.7 fractions. Equal *N*_max_ was observed in 5, 4, and 4 patients, respectively, while VMAT achieved a higher *N*_max_ in 3, 1, and 1 patients, respectively.

**FIGURE 2:**
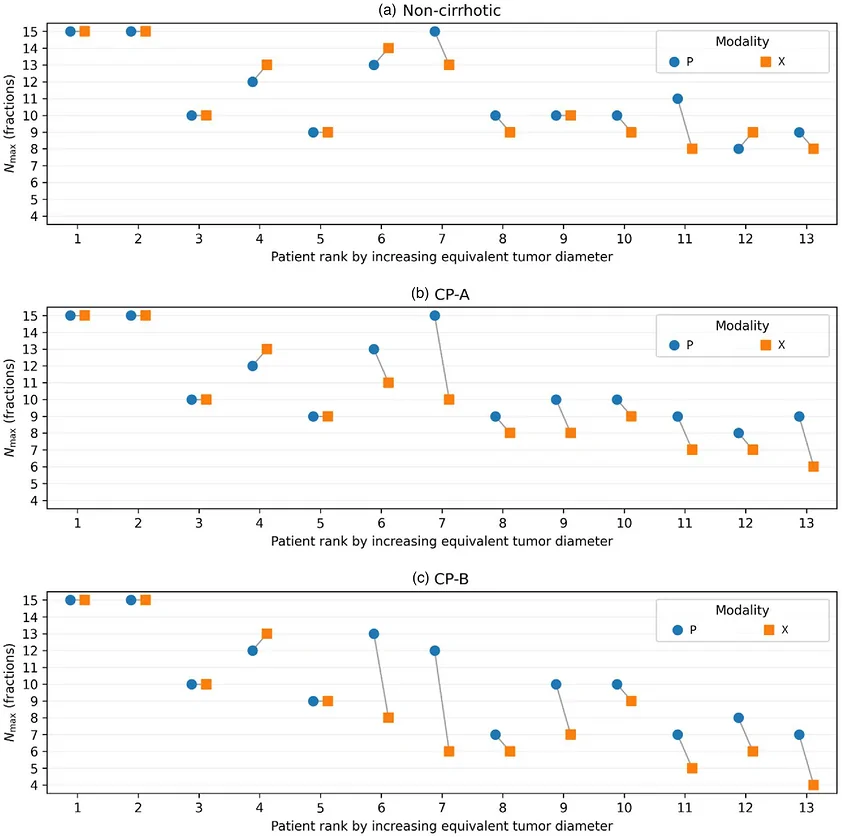
Nominal dose analysis: patient‐specific *N*_max_, ranked by increasing equivalent tumor diameter. P indicates IMPT; X indicates VMAT. *N*_max_ is the maximum number of fractions for which an acceptable plan could be achieved.

In the voxel‐wise maximum‐dose analysis, with MLD evaluated using the nominal dose, IMPT achieved a higher *N*_max_ than VMAT in 4 of 13, 7 of 13, and 8 of 13 patients under the non‐cirrhotic, CP‐A, and CP‐B assumptions, respectively (Figure 3). The mean ∆*N*_max_ was 0.0, 1.1, and 1.8 fractions for the corresponding liver‐function assumptions. Equal *N*_max_ was observed in 5, 5, and 4 patients, respectively, while VMAT achieved a higher *N*_max_ in 4, 1, and 1 patients, respectively.

**FIGURE 3:**
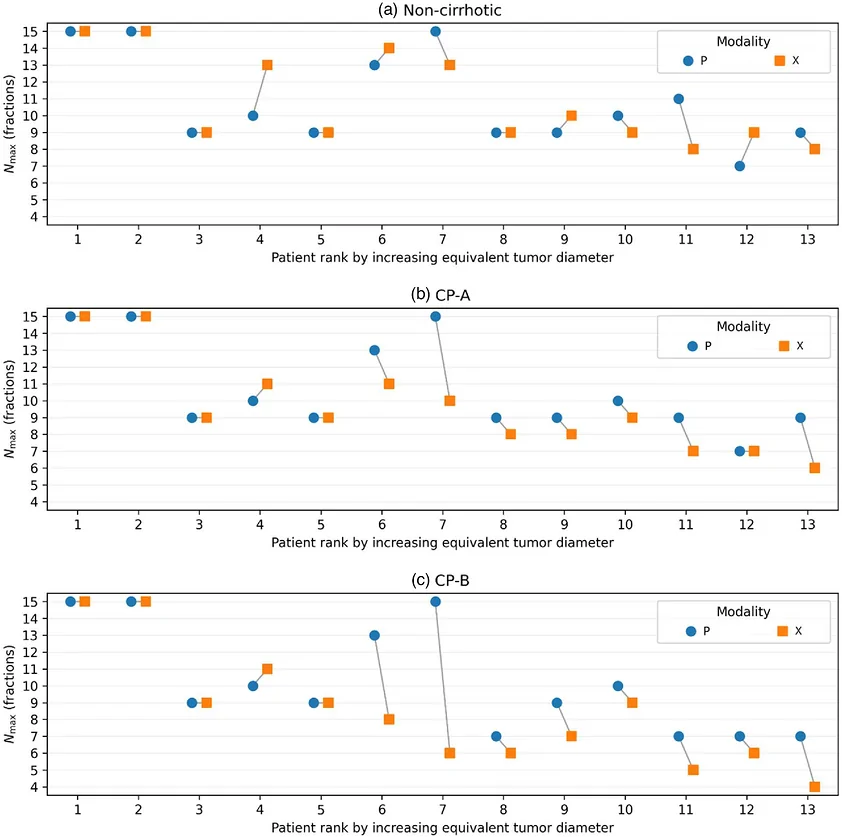
Voxel‐wise maximum‐dose analysis: patient‐specific *N*_max_, ranked by increasing equivalent tumor diameter. P indicates IMPT; X indicates VMAT. *N*_max_ is the maximum number of fractions for which an acceptable plan could be achieved.

### 3.2 Dose‐limiting factors

The distribution of recorded dose‐limiting factors differed between modalities (Figure 4). In the nominal‐dose analysis, IMPT plans were more frequently limited by GI tract maximum‐dose constraints, whereas VMAT plans were more frequently limited by MLD. This pattern was observed across all three liver‐function assumptions, with an increasing contribution of MLD among IMPT plans as the assumed liver baseline changed from non‐cirrhotic to CP‐B.

**FIGURE 4:**
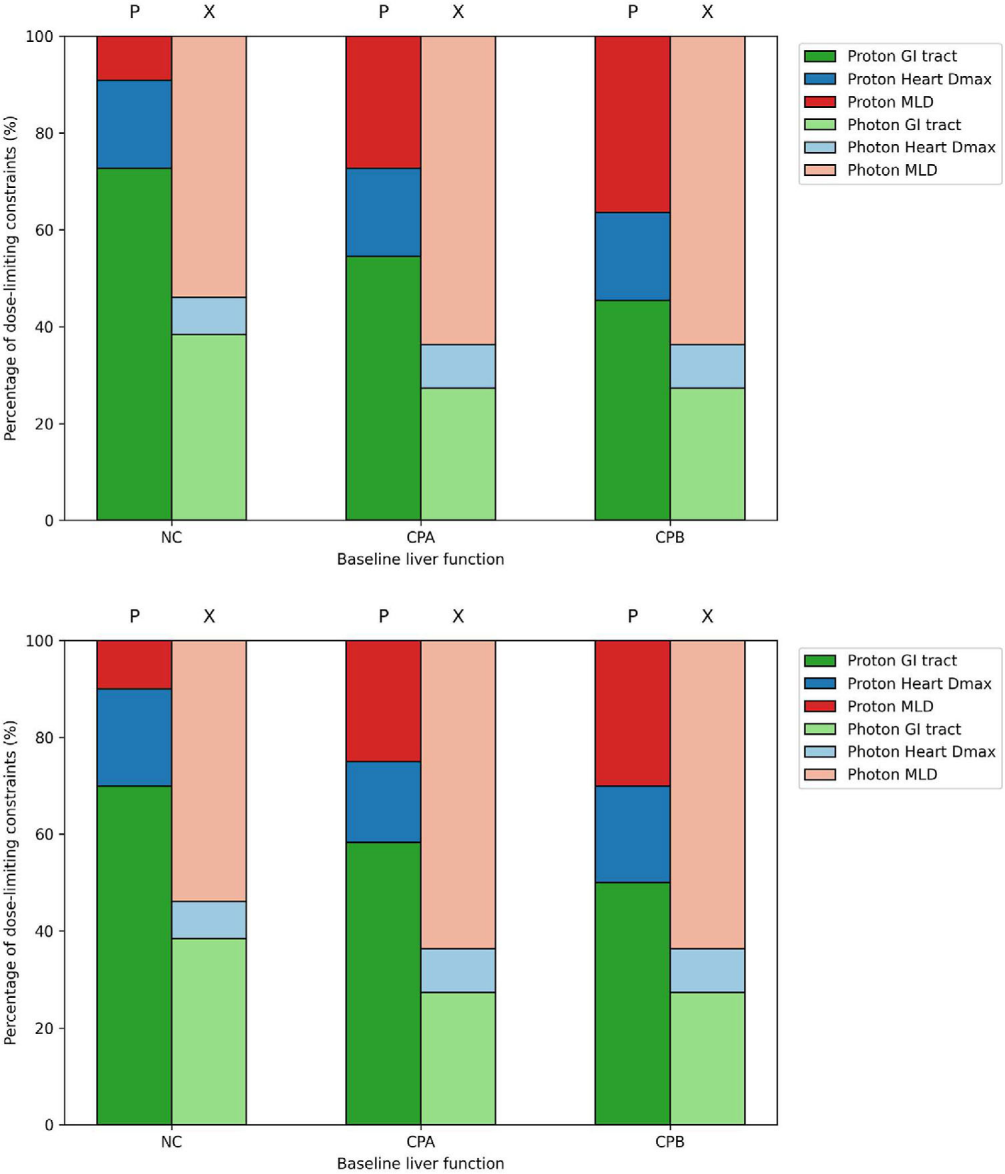
Percentage distribution of recorded dose‐limiting factors in nominal dose analysis (top) and voxel‐wise maximum dose analysis (bottom). P indicates IMPT; X indicates VMAT.

In the voxel‐wise maximum‐dose analysis, the same overall pattern was observed: IMPT dose escalation remained predominantly limited by GI tract maximum‐dose constraints, while VMAT dose escalation remained predominantly limited by MLD. Heart maximum dose was an infrequent limiting factor in both modalities and both analyses. The corresponding frequency summaries are provided in Tables S1 and S2.

### 3.3 Association between tumor size and IMPT‐VMAT difference in *N*_max_

The association between tumor size and ∆*N*_max_ is shown in Figures 5 and 6. In the nominal‐dose analysis, the Pearson correlation coefficient between equivalent tumor diameter and ∆ *N*_max_ was 0.27, 0.49, and 0.40 under the non‐cirrhotic, CP‐A, and CP‐B assumptions, respectively. The corresponding correlations using tumor volume were 0.17, 0.38, and 0.27. When grouped by equivalent tumor diameter, tumors smaller than 8 cm showed no average IMPT advantage, with a mean ∆ *N*_max_ of −0.25 fractions across all liver baselines. For tumors measuring 8–12 cm, the mean ∆*N*_max_ was 0.40, 2.00, and 3.00 fractions under the non‐cirrhotic, CP‐A, and CP‐B assumptions, respectively. For tumors larger than 12 cm, the corresponding mean ∆*N*_max_ values were 1.00, 1.75, and 2.00 fractions.

**FIGURE 5:**
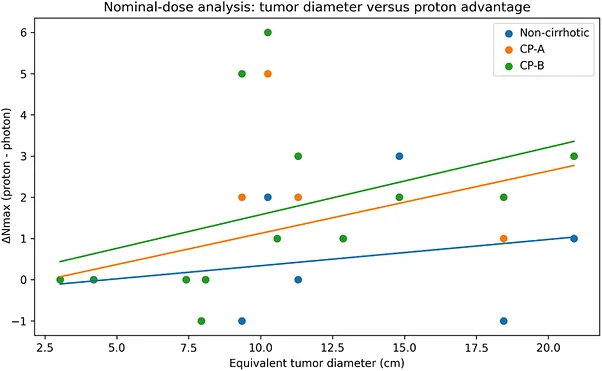
Nominal‐dose analysis: equivalent tumor diameter versus ∆ *N*_max_.

**FIGURE 6:**
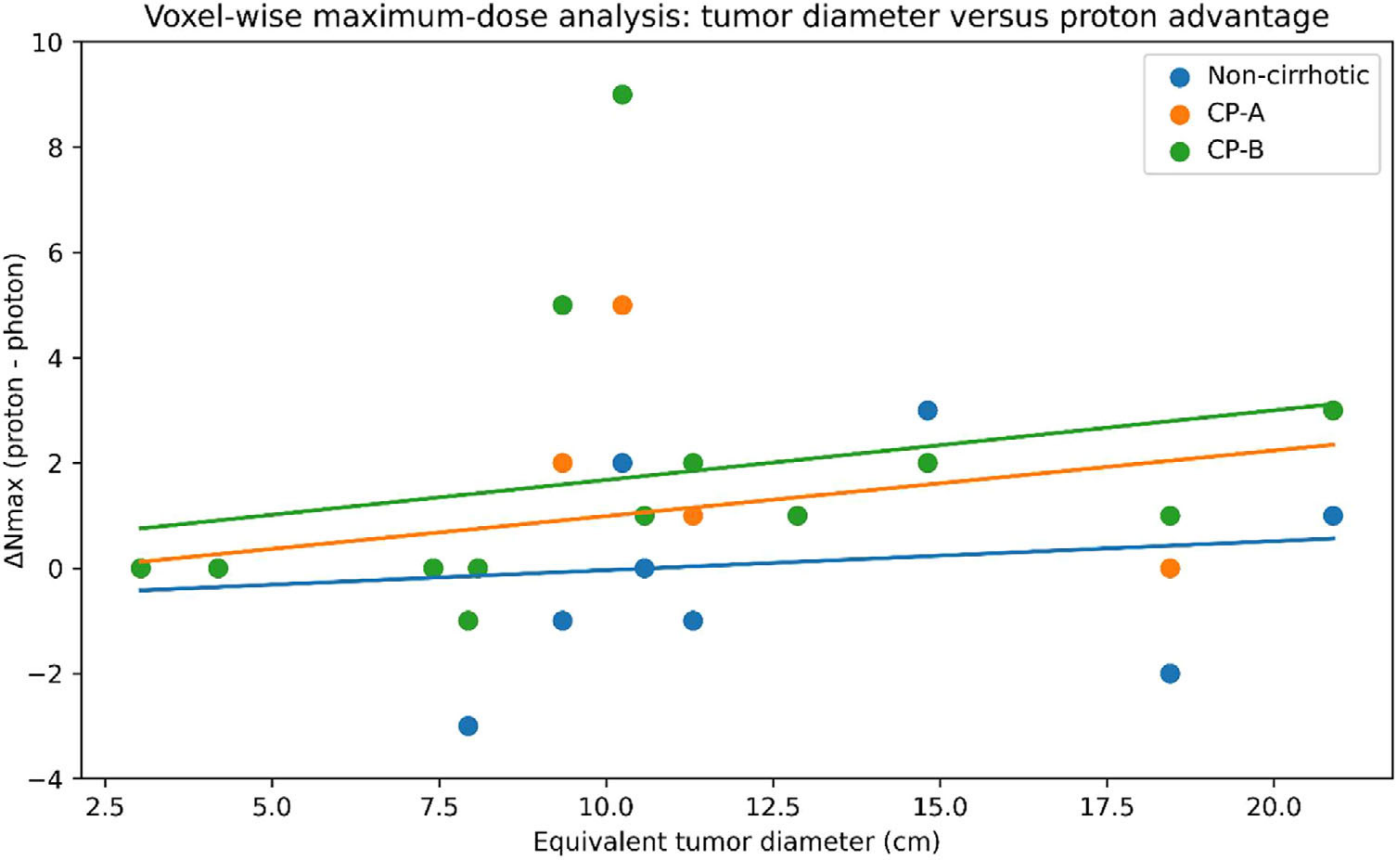
Voxel‐wise maximum‐dose analysis: equivalent tumor diameter versus ∆ *N*_max_.

In the voxel‐wise maximum‐dose analysis, the Pearson correlation coefficient between equivalent tumor diameter and ∆*N*_max_ was 0.18, 0.40, and 0.25 under the non‐cirrhotic, CP‐A, and CP‐B assumptions, respectively. The corresponding correlations using tumor volume were 0.13, 0.31, and 0.14. For tumors smaller than 8 cm, the mean ∆*N*_max_ was −0.75, −0.25, and −0.25 fractions under the non‐cirrhotic, CP‐A, and CP‐B assumptions, respectively. For tumors measuring 8–12 cm, the mean ∆*N_max_* was 0.00, 1.80, and 3.40 fractions. For tumors larger than 12 cm, the corresponding mean ∆*N*_max_ values were 0.75, 1.50, and 1.75 fractions.

### 3.4 Estimated reduction in RILD hazard using ULV/SLV

Among patients in whom IMPT achieved a higher *N*_max_, IMPT was also associated with lower estimated RILD hazard in most liver‐function assumptions. In the nominal‐dose analysis, the mean estimated RILD hazard reduction among patients with an IMPT *N*_max_ advantage was 80.2% using the male reference SLV and 84.7% using the female reference SLV under the non‐cirrhotic assumption. The corresponding reductions were 70.7% and 76.9% under the CP‐A assumption, and 35.5% and 38.9% under the CP‐B assumption.

In the voxel‐wise maximum‐dose analysis, the corresponding mean estimated RILD hazard reductions among patients with an IMPT *N*_max_ advantage were 84.0% and 87.7% under the non‐cirrhotic assumption, 73.3% and 79.1% under CP‐A, and 40.2% and 43.9% under CP‐B, using the male and female reference SLV assumptions, respectively.

Individual patient‐level ULV/SLV values and estimated RILD hazard reductions at modality‐specific *N*_max_ are provided in Table S3.

## 4 DISCUSSION

This study evaluated an isotoxic robust dose‐escalation framework for large HCCs near GI organs, comparing IMPT and VMAT under non‐cirrhotic, CP‐A, and CP‐B liver‐function assumptions. The principal finding was that the relative advantage of IMPT increased as hepatic reserve became more restrictive. In both nominal‐dose and voxel‐wise maximum‐dose analyses, the proton‐photon difference in achievable *N*_max_ was small under the non‐cirrhotic assumption but larger under CP‐A and CP‐B assumptions, indicating that the potential benefit of IMPT is greatest when photon escalation is limited by hepatic reserve.

The dose‐limiting patterns support this interpretation. VMAT escalation was predominantly limited by MLD, whereas IMPT escalation was more frequently limited by maximum dose to adjacent GI organs. This suggests that IMPT reduced the liver‐dose burden sufficiently to shift the limiting factor from a parallel‐organ liver constraint toward focal serial‐organ constraints. Therefore, the clinical value of IMPT in this setting depends on whether improved liver sparing can be translated into dose escalation without exceeding robust GI or heart maximum‐dose limits.

MLD remains the guideline‐based liver constraint for HCC radiotherapy and was therefore retained as the primary isotoxic escalation criterion.^4^ However, proton‐specific RILD studies suggest that ULV/SLV provides complementary information by quantifying preserved liver reserve.^5^, ^6^ In the present analysis, RILD hazard was evaluated at the respective modality‐specific *N*_max_, rather than at a fixed prescription level. Among patients with an IMPT *N*_max_ advantage, IMPT generally maintained higher ULV/SLV and lower estimated RILD hazard than VMAT despite escalation to a higher fraction number. This indicates that, in selected patients, the additional dose escalation achievable with IMPT did not fully consume its liver‐reserve advantage.

The relationship between tumor size and IMPT benefit was not monotonic. The association between equivalent tumor diameter and Δ*N*_max_ was stronger under the CP‐A and CP‐B assumptions than under the non‐cirrhotic assumption, consistent with increasing MLD limitation in VMAT as hepatic reserve became more restricted. However, the similar regression trends observed for the CP‐A and CP‐B groups suggest a saturation effect, whereby further proton benefit becomes increasingly constrained by adjacent serial organs and the upper tested fraction number rather than by liver dose. This explains why intermediate‐sized tumors showed the largest mean proton advantage, whereas very large tumors did not demonstrate proportionally greater benefit.

Importantly, the reduced Δ*N*_max_ observed in tumors larger than 12 cm did not indicate loss of the intrinsic liver‐sparing advantage of IMPT. At the respective modality‐specific maximum achievable prescription, IMPT continued to preserve higher ULV/SLV and lower estimated RILD hazard than VMAT (Table S3), indicating that, for very bulky tumors, the principal benefit of IMPT shifts from greater dose escalation toward better preservation of functional liver reserve.

Robustness evaluation materially affected the comparison. The mean IMPT advantage under the non‐cirrhotic assumption decreased when serial‐organ constraints were evaluated using voxel‐wise maximum dose, while advantages persisted under CP‐A and CP‐B assumptions. In the non‐cirrhotic analysis, two cases already favored VMAT under nominal evaluation (∆*N*_max_ = ‒1), but the difference widened to −3 and −2, respectively, when serial‐organ constraints were evaluated using voxel‐wise maximum dose. IMPT *N*_max_ decreased from 12 to 10 fractions in the first case and from 8 to 7 fractions in the second case, whereas VMAT remained unchanged at 13 and 9 fractions. Both IMPT plans were limited by maximum dose with the heart that was located within 5 mm from the CTV, indicating that setup and range uncertainties amplified focal dose to the adjacent serial organ. This demonstrates that nominal‐dose evaluation alone may overestimate dose escalation when steep gradients are placed near GI mucosa. For large HCCs close to stomach, duodenum, bowel, or heart, voxel‐wise maximum‐dose evaluation provides a more conservative and clinically relevant estimate of achievable escalation with proton, particularly in the non‐cirrhotic patient cohort.^17^

Several limitations should be acknowledged. This was a planning study in a limited cohort, and the RILD hazard estimates were based on published ULV/SLV hazard ratios rather than observed clinical toxicity. The analysis assumed reproducible breath hold during simulation and treatment, potentially supported by active breathing control or non‐invasive ventilation‐assisted breath hold. Therefore, the absolute *N*_max_ values are expected to depend on the motion‐management strategy, because residual motion directly affects setup uncertainty, robust target coverage, and near‐maximum dose to adjacent GI organs. Nevertheless, the relative escalation advantage of IMPT over VMAT, particularly under CP‐A and CP‐B assumptions, is expected to remain relevant because it is primarily driven by reduced uninvolved liver dose and preservation of ULV/SLV. The breath‐hold assumption is consistent with contemporary proton liver SBRT practice patterns reported in the recent NRG Oncology survey, which emphasized motion management, image guidance, and robustness handling of setup and range uncertainties for proton SBRT and hypofractionated liver treatments.^18^ The 5‐mm setup and 3% range uncertainty used in this study are therefore clinically representative, although delivered‐dose accumulation with daily anatomy, bowel motion, and adaptive replanning was not modeled. Finally, patient‐specific sex, height, and weight were unavailable, requiring reference SLV assumptions, and voxel‐wise maximum dose is conservative and may overestimate delivered focal dose if the worst‐case voxel dose is not realized consistently during treatment.^19^, ^20^, ^21^ In addition, although the same boundary‐search principle was applied to both modalities, the decision to perform targeted re‐optimization depended on planner assessment of whether a failed constraint was plausibly recoverable. The reported *N*_max_ should therefore be interpreted as the highest achievable fraction number under the applied planning strategy, rather than as a mathematically unique global maximum.

In summary, IMPT provided the greatest isotoxic escalation benefit when liver function was compromised and VMAT escalation was limited by MLD. However, the benefit was constrained by robust GI and heart maximum‐dose limits. These findings support a modality‐selection framework that integrates baseline liver function, MLD, ULV/SLV, tumor size, and robust serial‐organ dose, rather than relying on any single dosimetric endpoint.

## 5 CONCLUSIONS

IMPT provided greater isotoxic dose‐escalation potential than VMAT for selected patients with large HCC, with the greatest relative benefit observed under CP‐A and CP‐B liver‐function assumptions. The advantage resulted primarily from improved sparing of uninvolved liver, which shifted the dominant dose‐limiting factor from MLD with VMAT toward focal GI organ constraints with IMPT. Importantly, among patients with a higher IMPT *N*_max_, the proton plans generally retained a higher ULV/SLV and lower estimated RILD hazard at their respective escalated dose levels. Robust evaluation of serial‐organ dose materially affected the modality comparison and should be incorporated alongside baseline liver function and liver‐reserve metrics when selecting patients for proton therapy.

## AUTHOR CONTRIBUTIONS

Elise Cherek drafted the manuscript and performed data analysis. Mark Ka Heng Chan performed treatment planning, reviewed the final manuscript. Diane Schott performed the secondary evaluation and validation of the results data and reviewed the final manuscript.

## CONFLICT OF INTEREST STATEMENT

The authors declare that they have no known competing financial interests or personal relationships that could have appeared to influence the work reported in this paper.

## ETHICAL APPROVAL

Patient data were analyzed in a de‐identified manner in this retrospective study. The University of Nebraska Medical Center Office of Regulatory Affairs determined that this project does not constitute human subjects research under 45 CFR 46.102 because no interaction with individuals and no identifiable private information were involved. Therefore, institutional review board approval was not required.

## GENERATIVE AI STATEMENT

During the preparation of this work the author(s) used OpenAI's ChatGPT in order to improve linguistic and readability. After using this tool/service, the author(s) reviewed and edited the content as needed and take(s) full responsibility for the content of the publication.

## Supporting information




**Supporting Information**: acm270846‐sup‐0001‐TableS1.docx.

**Supporting Information**: acm270846‐sup‐0002‐TableS2.docx.

**Supporting Information**: acm270846‐sup‐0003‐TableS3.docx.

## Data Availability

Authors will share data upon request to the corresponding author.
