# Supplementary material for: Potential of isotoxic dose escalation with robustly optimized VMAT and IMPT for large hepatocellular carcinoma according to baseline liver function
Source: J Appl Clin Med Phys. 2026 Oct 6;27(10):e70846. doi: 10.1002/acm2.70846 (PMC13643040; doi:10.1002/acm2.70846)
Supplement: Supplementary file 2 [file ACM2-27-e70846-s002.docx]

**Supplementary Table S3. Patient-level ULV/SLV and RILD hazard reduction at modality-specific Nmax**

ULV/SLV values are reported using reference male and female SLV assumptions. Hazard reduction = 1 - 0.902^(Delta ULV/SLV percentage points), where Delta = IMPT - VMAT.

| **Scenario** | **Baseline** | **Patient #** | **Tumor volume (cc)** | **Nmax IMPT** | **Nmax VMAT** | **∆Nmax** | **IMPT ULV/SLV male** | **VMAT ULV/SLV male** | **HR IMPT/VMAT male** | **RILD hazard reduction male** | **IMPT ULV/SLV female** | **VMAT ULV/SLV female** | **HR IMPT/VMAT female** | **RILD hazard reduction female** |
| --- | --- | --- | --- | --- | --- | --- | --- | --- | --- | --- | --- | --- | --- | --- |
| Nominal | Non-cirrhotic | 1 | 14.56 | 15 | 15 | 0 | 20.8% | 14.5% | 0.524 | 47.6% | 25.7% | 17.9% | 0.450 | 55.0% |
| Nominal | Non-cirrhotic | 2 | 38.46 | 15 | 15 | 0 | 45.1% | 9.5% | 0.025 | 97.5% | 55.8% | 11.7% | 0.011 | 98.9% |
| Nominal | Non-cirrhotic | 3 | 213 | 10 | 10 | 0 | 34.2% | 9.5% | 0.079 | 92.1% | 42.2% | 11.8% | 0.043 | 95.7% |
| Nominal | Non-cirrhotic | 4 | 261.65 | 12 | 13 | -1 | 35.6% | 5.9% | 0.047 | 95.3% | 44.0% | 7.3% | 0.023 | 97.7% |
| Nominal | Non-cirrhotic | 5 | 275.97 | 9 | 9 | 0 | 48.0% | 14.0% | 0.030 | 97.0% | 59.3% | 17.3% | 0.013 | 98.7% |
| Nominal | Non-cirrhotic | 6 | 427.61 | 13 | 14 | -1 | 26.0% | 0.6% | 0.072 | 92.8% | 32.2% | 0.7% | 0.039 | 96.1% |
| Nominal | Non-cirrhotic | 7 | 562.22 | 15 | 13 | 2 | 20.9% | 3.2% | 0.161 | 83.9% | 25.9% | 4.0% | 0.105 | 89.5% |
| Nominal | Non-cirrhotic | 8 | 618.76 | 10 | 9 | 1 | 21.1% | 11.0% | 0.352 | 64.8% | 26.1% | 13.5% | 0.275 | 72.5% |
| Nominal | Non-cirrhotic | 9 | 755.12 | 10 | 10 | 0 | 35.1% | 8.0% | 0.061 | 93.9% | 43.4% | 9.9% | 0.031 | 96.9% |
| Nominal | Non-cirrhotic | 10 | 1114.75 | 10 | 9 | 1 | 45.4% | 7.3% | 0.020 | 98.0% | 56.1% | 9.0% | 0.008 | 99.2% |
| Nominal | Non-cirrhotic | 11 | 1701.43 | 11 | 8 | 3 | 22.5% | 14.9% | 0.456 | 54.4% | 27.8% | 18.4% | 0.378 | 62.2% |
| Nominal | Non-cirrhotic | 12 | 3283.46 | 8 | 9 | -1 | 27.1% | 8.7% | 0.149 | 85.1% | 33.5% | 10.7% | 0.095 | 90.5% |
| Nominal | Non-cirrhotic | 13 | 4768.5 | 9 | 8 | 1 | 71.8% | 16.2% | 0.003 | 99.7% | 88.7% | 20.0% | 0.001 | 99.9% |
| Nominal | CP-A | 1 | 14.56 | 15 | 15 | 0 | 20.8% | 14.5% | 0.524 | 47.6% | 25.7% | 17.9% | 0.450 | 55.0% |
| Nominal | CP-A | 2 | 38.46 | 15 | 15 | 0 | 45.1% | 9.5% | 0.025 | 97.5% | 55.8% | 11.7% | 0.011 | 98.9% |
| Nominal | CP-A | 3 | 213 | 10 | 10 | 0 | 34.2% | 9.5% | 0.079 | 92.1% | 42.2% | 11.8% | 0.043 | 95.7% |
| Nominal | CP-A | 4 | 261.65 | 12 | 13 | -1 | 35.6% | 5.9% | 0.047 | 95.3% | 44.0% | 7.3% | 0.023 | 97.7% |
| Nominal | CP-A | 5 | 275.97 | 9 | 9 | 0 | 48.0% | 14.0% | 0.030 | 97.0% | 59.3% | 17.3% | 0.013 | 98.7% |
| Nominal | CP-A | 6 | 427.61 | 13 | 11 | 2 | 26.0% | 12.5% | 0.248 | 75.2% | 32.2% | 15.5% | 0.178 | 82.2% |
| Nominal | CP-A | 7 | 562.22 | 15 | 10 | 5 | 20.9% | 15.1% | 0.551 | 44.9% | 25.9% | 18.7% | 0.479 | 52.1% |
| Nominal | CP-A | 8 | 618.76 | 9 | 8 | 1 | 24.8% | 16.2% | 0.412 | 58.8% | 30.6% | 20.0% | 0.335 | 66.5% |
| Nominal | CP-A | 9 | 755.12 | 10 | 8 | 2 | 35.1% | 23.1% | 0.289 | 71.1% | 43.4% | 28.5% | 0.215 | 78.5% |
| Nominal | CP-A | 10 | 1114.75 | 10 | 9 | 1 | 45.4% | 7.3% | 0.020 | 98.0% | 56.1% | 9.0% | 0.008 | 99.2% |
| Nominal | CP-A | 11 | 1701.43 | 9 | 7 | 2 | 31.9% | 22.3% | 0.371 | 62.9% | 39.4% | 27.5% | 0.294 | 70.6% |
| Nominal | CP-A | 12 | 3283.46 | 8 | 7 | 1 | 27.1% | 17.3% | 0.365 | 63.5% | 33.5% | 21.4% | 0.288 | 71.2% |
| Nominal | CP-A | 13 | 4768.5 | 9 | 6 | 3 | 71.8% | 48.5% | 0.091 | 90.9% | 88.7% | 59.9% | 0.051 | 94.9% |
| Nominal | CP-B | 1 | 14.56 | 15 | 15 | 0 | 20.8% | 14.5% | 0.524 | 47.6% | 25.7% | 17.9% | 0.450 | 55.0% |
| Nominal | CP-B | 2 | 38.46 | 15 | 15 | 0 | 45.1% | 9.5% | 0.025 | 97.5% | 55.8% | 11.7% | 0.011 | 98.9% |
| Nominal | CP-B | 3 | 213 | 10 | 10 | 0 | 34.2% | 9.5% | 0.079 | 92.1% | 42.2% | 11.8% | 0.043 | 95.7% |
| Nominal | CP-B | 4 | 261.65 | 12 | 13 | -1 | 35.6% | 5.9% | 0.047 | 95.3% | 44.0% | 7.3% | 0.023 | 97.7% |
| Nominal | CP-B | 5 | 275.97 | 9 | 9 | 0 | 48.0% | 14.0% | 0.030 | 97.0% | 59.3% | 17.3% | 0.013 | 98.7% |
| Nominal | CP-B | 6 | 427.61 | 13 | 8 | 5 | 26.0% | 24.5% | 0.848 | 15.2% | 32.2% | 30.2% | 0.816 | 18.4% |
| Nominal | CP-B | 7 | 562.22 | 12 | 6 | 6 | 27.7% | 31.0% | 1.409 | -40.9% | 34.3% | 38.4% | 1.528 | -52.8% |
| Nominal | CP-B | 8 | 618.76 | 7 | 6 | 1 | 32.2% | 26.6% | 0.566 | 43.4% | 39.7% | 32.9% | 0.495 | 50.5% |
| Nominal | CP-B | 9 | 755.12 | 10 | 7 | 3 | 35.1% | 30.6% | 0.630 | 37.0% | 43.4% | 37.9% | 0.565 | 43.5% |
| Nominal | CP-B | 10 | 1114.75 | 10 | 9 | 1 | 45.4% | 7.3% | 0.020 | 98.0% | 56.1% | 9.0% | 0.008 | 99.2% |
| Nominal | CP-B | 11 | 1701.43 | 7 | 5 | 2 | 41.3% | 37.2% | 0.650 | 35.0% | 51.1% | 45.9% | 0.587 | 41.3% |
| Nominal | CP-B | 12 | 3283.46 | 8 | 6 | 2 | 27.1% | 21.7% | 0.570 | 43.0% | 33.5% | 26.8% | 0.500 | 50.0% |
| Nominal | CP-B | 13 | 4768.5 | 7 | 4 | 3 | 88.1% | 80.8% | 0.470 | 53.0% | 108.9% | 99.8% | 0.393 | 60.7% |
| Voxmax | Non-cirrhotic | 1 | 14.56 | 15 | 15 | 0 | 20.8% | 14.5% | 0.524 | 47.6% | 25.7% | 17.9% | 0.450 | 55.0% |
| Voxmax | Non-cirrhotic | 2 | 38.46 | 15 | 15 | 0 | 45.1% | 9.5% | 0.025 | 97.5% | 55.8% | 11.7% | 0.011 | 98.9% |
| Voxmax | Non-cirrhotic | 3 | 213 | 9 | 9 | 0 | 36.7% | 14.5% | 0.101 | 89.9% | 45.3% | 17.9% | 0.059 | 94.1% |
| Voxmax | Non-cirrhotic | 4 | 261.65 | 10 | 13 | -3 | 40.1% | 5.9% | 0.029 | 97.1% | 49.6% | 7.3% | 0.013 | 98.7% |
| Voxmax | Non-cirrhotic | 5 | 275.97 | 9 | 9 | 0 | 48.0% | 14.0% | 0.030 | 97.0% | 59.3% | 17.3% | 0.013 | 98.7% |
| Voxmax | Non-cirrhotic | 6 | 427.61 | 13 | 14 | -1 | 26.0% | 0.6% | 0.072 | 92.8% | 32.2% | 0.7% | 0.039 | 96.1% |
| Voxmax | Non-cirrhotic | 7 | 562.22 | 15 | 13 | 2 | 20.9% | 3.2% | 0.161 | 83.9% | 25.9% | 4.0% | 0.105 | 89.5% |
| Voxmax | Non-cirrhotic | 8 | 618.76 | 9 | 9 | 0 | 24.8% | 11.0% | 0.241 | 75.9% | 30.6% | 13.5% | 0.172 | 82.8% |
| Voxmax | Non-cirrhotic | 9 | 755.12 | 9 | 10 | -1 | 40.0% | 8.0% | 0.037 | 96.3% | 49.4% | 9.9% | 0.017 | 98.3% |
| Voxmax | Non-cirrhotic | 10 | 1114.75 | 10 | 9 | 1 | 45.4% | 7.3% | 0.020 | 98.0% | 56.1% | 9.0% | 0.008 | 99.2% |
| Voxmax | Non-cirrhotic | 11 | 1701.43 | 11 | 8 | 3 | 22.5% | 14.9% | 0.456 | 54.4% | 27.8% | 18.4% | 0.378 | 62.2% |
| Voxmax | Non-cirrhotic | 12 | 3283.46 | 7 | 9 | -2 | 29.7% | 8.7% | 0.114 | 88.6% | 36.7% | 10.7% | 0.069 | 93.1% |
| Voxmax | Non-cirrhotic | 13 | 4768.5 | 9 | 8 | 1 | 71.8% | 16.2% | 0.003 | 99.7% | 88.7% | 20.0% | 0.001 | 99.9% |
| Voxmax | CP-A | 1 | 14.56 | 15 | 15 | 0 | 20.8% | 14.5% | 0.524 | 47.6% | 25.7% | 17.9% | 0.450 | 55.0% |
| Voxmax | CP-A | 2 | 38.46 | 15 | 15 | 0 | 45.1% | 9.5% | 0.025 | 97.5% | 55.8% | 11.7% | 0.011 | 98.9% |
| Voxmax | CP-A | 3 | 213 | 9 | 9 | 0 | 36.7% | 14.5% | 0.101 | 89.9% | 45.3% | 17.9% | 0.059 | 94.1% |
| Voxmax | CP-A | 4 | 261.65 | 10 | 11 | -1 | 40.1% | 14.7% | 0.072 | 92.8% | 49.6% | 18.1% | 0.039 | 96.1% |
| Voxmax | CP-A | 5 | 275.97 | 9 | 9 | 0 | 48.0% | 14.0% | 0.030 | 97.0% | 59.3% | 17.3% | 0.013 | 98.7% |
| Voxmax | CP-A | 6 | 427.61 | 13 | 11 | 2 | 26.0% | 12.5% | 0.248 | 75.2% | 32.2% | 15.5% | 0.178 | 82.2% |
| Voxmax | CP-A | 7 | 562.22 | 15 | 10 | 5 | 20.9% | 15.1% | 0.551 | 44.9% | 25.9% | 18.7% | 0.479 | 52.1% |
| Voxmax | CP-A | 8 | 618.76 | 9 | 8 | 1 | 24.8% | 16.2% | 0.412 | 58.8% | 30.6% | 20.0% | 0.335 | 66.5% |
| Voxmax | CP-A | 9 | 755.12 | 9 | 8 | 1 | 40.0% | 23.1% | 0.175 | 82.5% | 49.4% | 28.5% | 0.116 | 88.4% |
| Voxmax | CP-A | 10 | 1114.75 | 10 | 9 | 1 | 45.4% | 7.3% | 0.020 | 98.0% | 56.1% | 9.0% | 0.008 | 99.2% |
| Voxmax | CP-A | 11 | 1701.43 | 9 | 7 | 2 | 31.9% | 22.3% | 0.371 | 62.9% | 39.4% | 27.5% | 0.294 | 70.6% |
| Voxmax | CP-A | 12 | 3283.46 | 7 | 7 | 0 | 29.7% | 17.3% | 0.280 | 72.0% | 36.7% | 21.4% | 0.207 | 79.3% |
| Voxmax | CP-A | 13 | 4768.5 | 9 | 6 | 3 | 71.8% | 48.5% | 0.091 | 90.9% | 88.7% | 59.9% | 0.051 | 94.9% |
| Voxmax | CP-B | 1 | 14.56 | 15 | 15 | 0 | 20.8% | 14.5% | 0.524 | 47.6% | 25.7% | 17.9% | 0.450 | 55.0% |
| Voxmax | CP-B | 2 | 38.46 | 15 | 15 | 0 | 45.1% | 9.5% | 0.025 | 97.5% | 55.8% | 11.7% | 0.011 | 98.9% |
| Voxmax | CP-B | 3 | 213 | 9 | 9 | 0 | 36.7% | 14.5% | 0.101 | 89.9% | 45.3% | 17.9% | 0.059 | 94.1% |
| Voxmax | CP-B | 4 | 261.65 | 10 | 11 | -1 | 40.1% | 14.7% | 0.072 | 92.8% | 49.6% | 18.1% | 0.039 | 96.1% |
| Voxmax | CP-B | 5 | 275.97 | 9 | 9 | 0 | 48.0% | 14.0% | 0.030 | 97.0% | 59.3% | 17.3% | 0.013 | 98.7% |
| Voxmax | CP-B | 6 | 427.61 | 13 | 8 | 5 | 26.0% | 24.5% | 0.848 | 15.2% | 32.2% | 30.2% | 0.816 | 18.4% |
| Voxmax | CP-B | 7 | 562.22 | 15 | 6 | 9 | 27.7% | 31.0% | 1.409 | -40.9% | 34.3% | 38.4% | 1.528 | -52.8% |
| Voxmax | CP-B | 8 | 618.76 | 7 | 6 | 1 | 32.2% | 26.6% | 0.566 | 43.4% | 39.7% | 32.9% | 0.495 | 50.5% |
| Voxmax | CP-B | 9 | 755.12 | 9 | 7 | 2 | 40.0% | 30.6% | 0.382 | 61.8% | 49.4% | 37.9% | 0.305 | 69.5% |
| Voxmax | CP-B | 10 | 1114.75 | 10 | 9 | 1 | 45.4% | 7.3% | 0.020 | 98.0% | 56.1% | 9.0% | 0.008 | 99.2% |
| Voxmax | CP-B | 11 | 1701.43 | 7 | 5 | 2 | 41.3% | 37.2% | 0.650 | 35.0% | 51.1% | 45.9% | 0.587 | 41.3% |
| Voxmax | CP-B | 12 | 3283.46 | 7 | 6 | 1 | 29.7% | 21.7% | 0.438 | 56.2% | 36.7% | 26.8% | 0.360 | 64.0% |
| Voxmax | CP-B | 13 | 4768.5 | 7 | 4 | 3 | 88.1% | 80.8% | 0.470 | 53.0% | 108.9% | 99.8% | 0.393 | 60.7% |

**Abbreviations:** ULV, uninvolved liver volume receiving <1 Gy; SLV, standard liver volume; RILD, radiation-induced liver disease; HR, hazard ratio; IMPT, intensity-modulated proton therapy; VMAT, volumetric-modulated arc therapy; CP-A, Child-Pugh A; CP-B, Child-Pugh B.
