# Supplementary material for: Potential of isotoxic dose escalation with robustly optimized VMAT and IMPT for large hepatocellular carcinoma according to baseline liver function
Source: J Appl Clin Med Phys. 2026 Oct 6;27(10):e70846. doi: 10.1002/acm2.70846 (PMC13643040; doi:10.1002/acm2.70846)
Supplement: Supplementary file 3 [file ACM2-27-e70846-s003.docx]

**Table S1.** Frequency summary of recorded dose-limiting factors in the nominal-dose analysis. Multiple recorded categories for the same patient were counted separately.

| **Baseline** | **Modality** | **GI tract** | **Heart Dmax** | **MLD** | **Total recorded constraints** |
| --- | --- | --- | --- | --- | --- |
| Non-cirrhotic | IMPT | 8 | 2 | 1 | 11 |
| Non-cirrhotic | VMAT | 5 | 1 | 7 | 13 |
| CP-A | IMPT | 6 | 2 | 3 | 11 |
| CP-A | VMAT | 3 | 1 | 7 | 11 |
| CP-B | IMPT | 5 | 2 | 4 | 11 |
| CP-B | VMAT | 3 | 1 | 7 | 11 |
